# Gene set enrichment meta-learning analysis: next- generation sequencing versus microarrays

**DOI:** 10.1186/1471-2105-11-176

**Published:** 2010-04-08

**Authors:** Gregor Stiglic, Mateja Bajgot, Peter Kokol

**Affiliations:** 1 Faculty of Health Sciences, University of Maribor, Zitna ulica 15, 2000 Maribor, Slovenia

## Abstract

**Background:**

Reproducibility of results can have a significant impact on the acceptance of new technologies in gene expression analysis. With the recent introduction of the so-called next-generation sequencing (NGS) technology and established microarrays, one is able to choose between two completely different platforms for gene expression measurements. This study introduces a novel methodology for gene-ranking stability analysis that is applied to the evaluation of gene-ranking reproducibility on NGS and microarray data.

**Results:**

The same data used in a well-known MicroArray Quality Control (MAQC) study was also used in this study to compare ranked lists of genes from MAQC samples A and B, obtained from Affymetrix HG-U133 Plus 2.0 and Roche 454 Genome Sequencer FLX platforms. An initial evaluation, where the percentage of overlapping genes was observed, demonstrates higher reproducibility on microarray data in 10 out of 11 gene-ranking methods. A gene set enrichment analysis shows similar enrichment of top gene sets when NGS is compared with microarrays on a pathway level. Our novel approach demonstrates high accuracy of decision trees when used for knowledge extraction from multiple bootstrapped gene set enrichment analysis runs. A comparison of the two approaches in sample preparation for high-throughput sequencing shows that alternating decision trees represent the optimal knowledge representation method in comparison with classical decision trees.

**Conclusions:**

Usual reproducibility measurements are mostly based on statistical techniques that offer very limited biological insights into the studied gene expression data sets. This paper introduces the meta-learning-based gene set enrichment analysis that can be used to complement the analysis of gene-ranking stability estimation techniques such as percentage of overlapping genes or classic gene set enrichment analysis. It is useful and practical when reproducibility of gene ranking results or different gene selection techniques is observed. The proposed method reveals very accurate descriptive models that capture the co-enrichment of gene sets which are differently enriched in the compared data sets.

## Background

DNA microarray technology has extended to all fields of genomic research and has become practically the primary tool for gene expression analysis [1]. Significant biotechnological advances changed that prospective and, with the recent introduction of the so-called next-generation sequencing (NGS) technology, a completely different platform for gene expression measurement has emerged. With the development of NGS technology, it became possible to analyze gene expression by direct shotgun sequencing of complementary DNA synthesized from RNA samples [2,3]. The new technology rapidly became very popular mainly because of the enormous time and cost savings, which could enable a massive throughput in the gathering of genomic data. Moreover, while earlier techniques remain very expensive, NGS has the potential to make genome sequencing a routine medical diagnostic procedure. In spite of all advantages, there are certain aspects that need to be explored before the NGS technology can be widely applied in gene expression analysis. As a tool for gene expression analysis, NGS technologies need to provide reliable gene expression data. Additionally, one should be able to assess the reproducibility of results from the statistical and biological points of view.

Ma [4] wrote one of the first papers in gene expression analysis, comparing different supervised gene selection methods by bootstrapping the samples of the initial data set. Ma measured the concordance and reproducibility of the supervised gene screening based on eight different gene selection methods. The measurements of concordance were done by overlapping the selected genes with different settings for n top genes. Among other conclusions, this empirical study once again explained that rankings of genes that pass through different gene selection methods may be considerably different. Another similar study, conducted by Qiu et al. [5], evaluated the stability of differentially expressed genes using the measurement of frequency, by which a given gene is selected across subsamples. They showed that re-sampling can be an appropriate technique to determine a set of genes with sufficiently high frequency. Furthermore, they recommended using re-sampling techniques to assess the variability of different performance indicators.

The goal of the recent large reproducibility study named Microarray Quality Control (MAQC) Project [6] was to measure and evaluate the differences between most popular microarray platforms. The authors of the MAQC study have used a simple and effective reproducibility metric called percentage of overlapping genes, simply called POG score. They concluded that a fold change-based method showed the most reproducible results when intra-platform reproducibility for differently expressed genes was measured using the POG score. Samples A and B from MAQC study were recently used by Mane et al. [7] to perform deep sequencing using massively parallel sequencing. Their study focused on technical reproducibility and mapping of reads to individual RefSeq genes. Using MAQC metrics in evaluating the performance of gene expression platforms, they observed excellent reproducibility, sensitivity, and specificity of the NGS platform. Data from both studies represent the appropriate material for demonstration of our proposed meta-learning-based gene set enrichment analysis.

Our study focuses on the comparison of gene ranking result reproducibility, using simple stability metrics and a more advanced pathway level of comparison of results obtained from microarray and NGS platforms. In addition to empirical evaluation, we propose a novel gene set enrichment-based analysis methodology that can significantly facilitate the process of gene set enrichment analysis when one intends to compare results of different studies, platforms, or even gene-ranking methods. It has to be noted that, in contrast to Mane et al. and the original MAQC study, where technical reproducibility of gene expression measurement is observed, this study focuses on reproducibility of gene-ranking results, sometimes also referred to as gene- ranking stability.

## Methods

### Microarray Data Sets

Our study used microarray data from Affymetrix data sets that were used in the MAQC study. This platform was chosen due to the high number of test sites (six data sets), allowing more accurate results of reproducibility and pathway-level analysis. MAQC CEL file data were analyzed with BioConductor to generate probeset-level data using the justPlier() function. Probe-level data were quantile-normalized before PLIER summarization per test site. An offset value of 16 was added to each probeset-level data point. All six normalized data sets, each containing five replicates of sample A (pooled human cell lines) compared with sample B (pooled human brain), were obtained from the official MACQ website.

Affymetrix HG-U133 Plus 2.0 GeneChip probe ids were collapsed into gene symbols using maximal expression for multiple probes mapped to a single gene symbol. This step reduced the dimensionality of data sets from the original 54,675 probes to 20,647 gene symbols.

### Next-generation Sequencing Data Sets

Two data sets from a recent paper by Mane et al. were used to compare the reproducibility of microarray versus NGS data sets. Deep sequencing of the MAQC reference RNA samples was done using Roche's 454 Genome Sequencer FLX (GS FLX). More than 3.6 million sequence reads with an average length of 250 bp were generated for cDNA from the MAQC A and B samples. Using RefSeq database, 64% of all reads could be matched to annotated genes using BLAST. Following mapping to RefSeq IDs, so-called digital gene expression can be measured by counting the numbers of reads that map to individual genes. A supplementary table presenting counts for all mapped reads from the paper by Mane et al. was used to compare the NGS and microarray data in this study. It contains hit counts for the sequencing runs for the A and B samples processed using either the Transcriptome Sequencing (TSEQ) or Oligo DT (ODT) protocols. The data were divided into two data sets- ODT and TSEQ. There were 10 samples (five from MAQC A sample and five from MAQC B sample) in the ODT data set and 12 samples (MAQC B sample was sequenced seven times) in the TSEQ dataset. An initial pool of 24,655 RefSeq symbols was mapped to 16,578 gene symbols for compatibility with microarray data sets, especially for pathway-based analysis.

The ODT and TSEQ cDNA sample preparation methods were introduced by Mane et al in their paper comparing more technical aspects of NGS to microarrays. The TSEQ method used random primers and had to be applied to heat-fragmented mRNA strands to generate a single-stranded cDNA library for sequencing using the standard Roche GC Amplicon sequencing procedure. The random primers were used to remove a potential 3' bias in the ODT preparation. On the other hand, the ODT preparation method was used to prepare double-stranded cDNA for the standard Roche GS DNA Library preparation and sequencing procedure. To eliminate reads with long poly A strings, modified oligo dT primers ending with two different additional nucleotides were used in some sequencing runs for this method. Furthermore, both methods required a thorough depletion of rRNA, which can constitute as much as 98% of the total RNA, to minimize the number of sequencing reads from rRNA contamination. After multiple-reduction steps, the rRNA reads were finally reduced to less than 10%. General information on data sets used in this study is summarized in Table 1.

**Table 1:** Basic information on MAQC sample A vs B data sets

Name	Platform	Number of A/B samples	Expression measurements	Common mapped genes
AFX 1	Affymetrix HG-U133 Plus 2.0	5/5	54,675	15,578
AFX 2	Affymetrix HG-U133 Plus 2.0	5/5	54,675	15,578
AFX 3	Affymetrix HG-U133 Plus 2.0	5/5	54,675	15,578
AFX 4	Affymetrix HG-U133 Plus 2.0	5/5	54,675	15,578
AFX 5	Affymetrix HG-U133 Plus 2.0	5/5	54,675	15,578
AFX 6	Affymetrix HG-U133 Plus 2.0	5/5	54,675	15,578
TSEQ	Roche 454 Genome Sequencer	5/7	24,655	15,578
ODT	Roche 454 Genome Sequencer	5/5	24,655	15,578

### Percentage of Overlapping Genes

Reproducibility of experiments is one of the most important measures to consider when different gene expression analysis platforms are compared. This study used the so-called percentage of overlapping genes (POG) metric that was already proposed in the original MAQC study. POG can be calculated from two lists of ranked genes that are of equal length. It is calculated as the number of genes in common divided by the number of genes in each of the two equal-length lists. Usually, all available genes are ranked and POG is calculated for sublists of different lengths. Results of such comparison are the most suitable for visual representation.

In our study, only gene symbols present in both compared platforms (13,632 common genes) were used to allow an objective comparison of POG score. Initially, k subsets of original data sets were created using sampling with replacement, also called bootstrapping. Eleven gene-ranking methods from the Bioconductor package GeneSelector by Boulesteix and Slawski [8] were used to construct the original list of ranked genes *l*_*o *_and k ranked lists *l*_1_, *l*_2_, ..., *l*_*k *_of genes on each subset. Average POG score *POG*_*avg *_was measured by averaging all pairwise comparisons of *k *ranked gene lists with the original ranking on the initial data set.

Altogether, POG scores were calculated using the 11 gene selection methods from GeneSelector summarized in Table 2. The R code for the POG score experiment is available in Additional file 1.

**Table 2:** Gene selection methods used in calculating percentage of overlapping genes

Selection method	Short name	Reference
T-statistic	TTest	Boulesteix and Slawski, 2009 [8]
Fold change	FC	Boulesteix and Slawski, 2009 [8]
Wilcoxon statistic	Wilcoxon	Boulesteix and Slawski, 2009 [8]
Welch T-statistic	WelchT	Boulesteix and Slawski, 2009 [8]
Bayesian t-statistic 1	BaldiLong	Baldi and Long, 2001 [9]
Bayesian t-statistic 2	FoxDimmic	Fox and Dimmic, 2006 [10]
Shrinkage t-statistic	ShrinkageT	Opgen-Rhein and Strimmer, 2007 [11]
Soft-threshold t-statistic	SoftthresholdT	Wu, 2005 [12]
Parametric empirical Bayes	Limma	Smyth, 2004 [13]
Nonparametric empirical Bayes	Ebam	Efron et al., 2001 [14]
Permutation test	Permutation	

### Measuring Gene Set Enrichment

Current high-throughput-based studies usually generate large lists of differently expressed genes as their outputs. However, the biological interpretation of such lists (ranging in size from hundreds to thousands of genes) is still a challenging task. Over the last few decades, bioinformatics specialists have collected a wide spectrum of biological knowledge that is deposited in public databases and scientific papers. It is therefore possible to assemble a summary of genes that are present in similar clinical conditions in a collection of gene sets that can be used for so-called functional analysis. A number of high-throughput enrichment tools, like Onto-Express [15], MAPPFinder [16], GoMiner [17], DAVID [18], and others were developed in initial studies to help scientists do a functional analysis of large gene lists. Gene set analysis is also used to eliminate the effect of generally low overlaps between different microarray data sets or platforms [19]. For specific details and an exhaustive coverage of similar techniques, the reader is advised to consult the review papers by Huang et al. [20], Dinu et al. [21], or Song et al. [22].

This study employed a widely used tool for gene set enrichment, simply called gene set enrichment analysis. An earlier version of this approach, also called gene set enrichment analysis, has been previously described by Lamb et al. [23] and Mootha et al. [24]. Their technique was extended by Sweet-Cordero et al. [25] to allow the analysis of multiple gene sets as well as multiple data sets. A refinement of the GSEA methodology with a broader applicability along several kinds of data sets has been developed by Subramanian et al. [26]. Implementation of GSEA by Subramanian et al. developed at Broad Institute is also available as open-source project written in Java. This was one of the reasons it was selected for gene set enrichment analysis in our study where it was necessary to adapt the current implementation to the needs of the proposed meta-learning-based enrichment analysis.

An analysis of gene set enrichment using GSEA application was performed on each of the eight data sets as described in [26]. As recommended by the GSEA authors, gene set permutation was used instead of phenotype permutation due to the small sample size. Original data set ids were collapsed into gene symbols before GSEA was run. For easier reproducibility of results, the same permutation random seed (149) was used in all GSEA runs. Based on POG score results, where it produced the most stable lists of ranked genes, fold change was also the method used for gene selection in GSEA to compare pathway analysis results between NGS and microarray-based gene expression analysis. MSigDB C2 v2.5 gene sets database [27] was used to evaluate 1410 gene sets after short (< 15) and long (> 500) gene lists were removed.

### Meta-Learning Analysis

Our proposed meta-learning-based GSEA originates from the idea to automate comparisons of multiple GSEA results that have to be done manually. The novel approach is called gene set enrichment meta-learning analysis (GSE-MLA), inspired by the meta-learning theory [28]. By definition, this subfield of machine learning introduces the term meta-data that is used to derive meta-knowledge from the results of the studied algorithm. In our case, GSEA is the source of meta-data that is represented as normalized enrichment scores (NES) measured for gene sets of interest. Different supervised machine learning methods can be further applied to the meta-data to capture the knowledge. Of course, it is very important that such models represent extracted knowledge in comprehensible form. Our study used decision tree algorithms for the interpretation of meta-knowledge and visualization of significant patterns that are characteristic of the compared gene expression analysis platforms. In other words, the GSE-MLA tries to extract and visualize the knowledge describing the characteristics of GSEA when run on microarray or NGS data sets.

**Figure 1:**
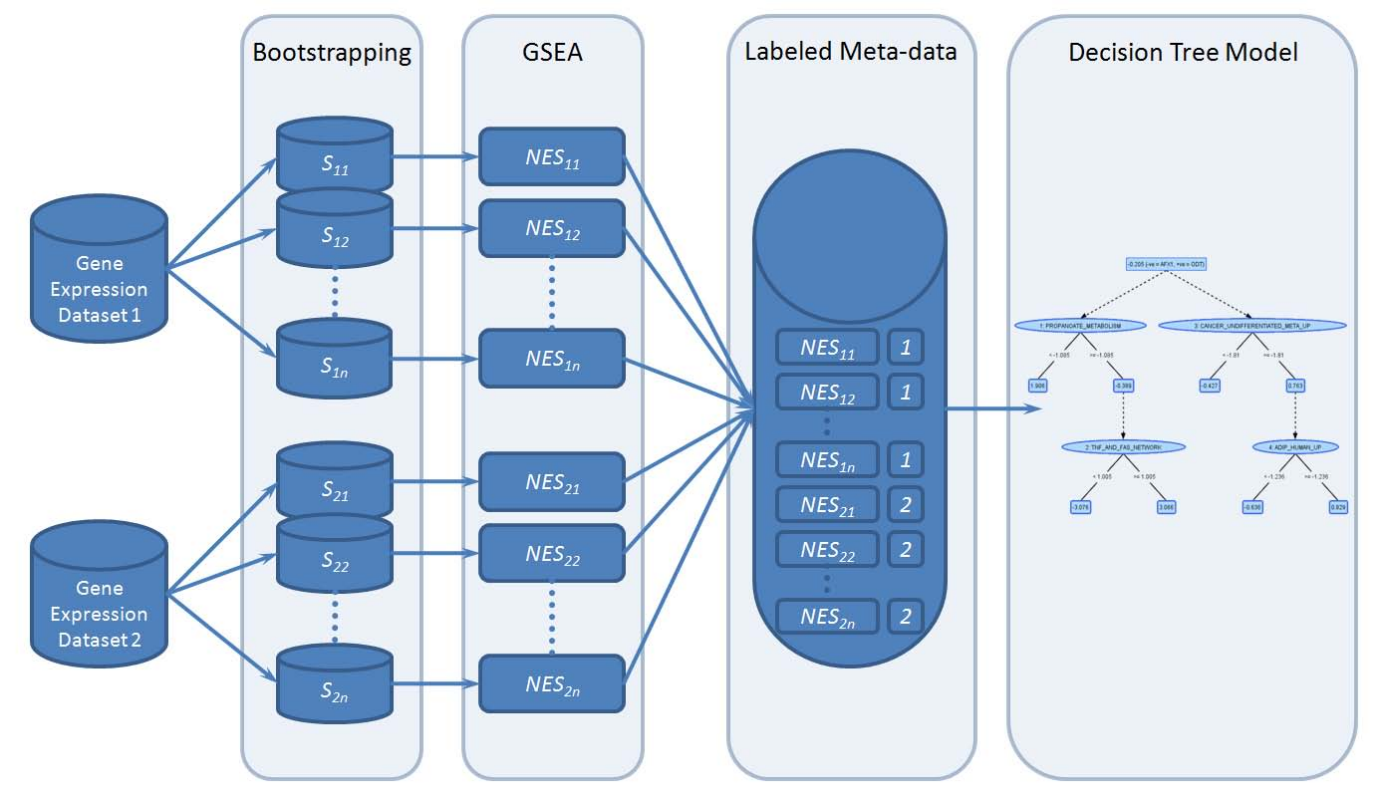
**GSE-MLA workflow**. Workflow of the GSE-MLA procedure describing the process from the initial data sets (e.g. next-generation sequencing vs. microarrays) to the final decision tree model.

From a technical point of view, one needs enough meta-data samples to build a reliably supervised classification model. In our study, GSEA was run 100 times on bootstrapped samples for each of two compared original data sets. Each sample, containing NES measurements for all observed gene sets where FDR <25%, was labeled according to the data set of origin and deposited in a meta-learning data set. This data set was then used to build a final knowledge representation model that was built using 200 meta-data samples. Figure 1 presents a diagram explaining the GSE-MLA workflow from the initial data set to the final decision tree model. However, it is also possible to analyze the results from GSE-MLA purely statistically instead of building a decision tree model. A simple statistical test like the student t-test could also be used to rank the gene sets and observe their ability to separate the two observed collections of meta-data.

The ability to track and evaluate every step in the decision-making process is the most important factor for trusting the decisions gained from data-mining methods. Examples of such techniques are decision trees that possess an important advantage in comparison with competitive classification methods-i.e., the symbolic representation of the extracted knowledge. Decision trees, along with rule-based classifiers, represent a group of classifiers that perform classification by a sequence of simple, easy-to-understand tests whose semantics are intuitively clear to domain experts [29]. Data analysis described in this paper was performed using libraries from Weka machine learning environment [30]. Two classical decision tree-building techniques (J48 [31] and SimpleCART [32]), along with an advanced alternating decision tree (ADTree [33]), were used to build decision tree models.

While J48 and SimpleCART represent two classical decision tree-building techniques that were widely used in the past, ADTree deserves a short introduction. It is an advanced decision tree-building technique based on boosting [34] algorithms that are usually used when ensembles of classifiers [35] are built. In this specific case, boosting is used to "boost" the extraction of knowledge in the form of separate branches in a decision tree. Therefore, the complexity of decision tree interpretation is higher, but, on the other hand, ADTree also performs much better in terms of classification accuracy and other performance metrics.

The performance of each decision tree built during this study was evaluated by measuring the classification accuracy (ACC) and area under ROC curve (AUC) metrics. Cross-validation with ten folds was used to calculate both performance metrics. J48 and SimpleCART trees were used with default Weka parameters, while the number of ADTree boosting iterations was lowered from 10 to 5 to allow better comprehensibility of the built models. Our empirical results showed no significant loss of ACC or AUC when using only five boosting iterations.

The pseudo-code of the algorithm used is summarized below:

1) Repeat the bootstrapping of the samples from each of the two compared gene expression data sets (GED) *n *times.

2) For each of the *2n *bootstrapped data sets, calculate normalized enrichment scores (NES) for all gene sets.

3) Label NES vectors by their origin (GED1 or GED2).

4) Build comprehensible classification model using all *2n *labeled NES vectors (decision tree is recommended).

In addition to knowledge extraction and classification performance evaluation, GSE-MLA results can be used as input data for GeneSelector to assess overlap of gene sets between different data sets. In our case, we can measure the POGS between gene set enrichment analysis on microarray versus NGS data sets.

## Results

### Gene Ranking Stability

**Figure 2:**
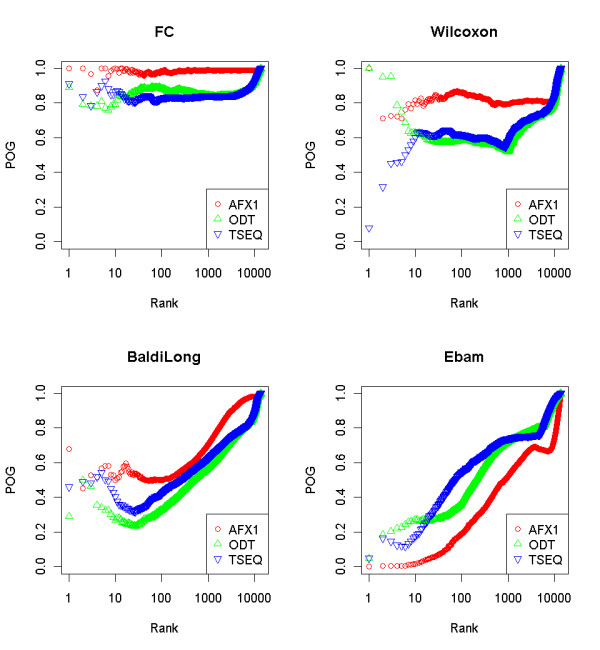
**POG scores**. Comparison of POG scores for microarray AFX1 (red), ODT (green), and TSEQ (blue) data sets.

In the initial experiment, we tried to find the gene ranking method with the highest reproducibility score. It is important to check whether NGS data follow similar characteristics to microarray data sets (the MAQC study determined that simple fold change ranking guarantees the highest reproducibility scores). To our knowledge, there is no similar study that would evaluate the stability of NGS of MAQC data using the POG metric. Therefore, POG was calculated for 11 gene selection methods implemented in GeneSelector. Altogether, there are 15 gene selection methods, but due to technical reasons (mainly the small number of samples), we were not able to run all of them on our data. Figure 2 represents initial results from affymetrix (AFX1) and two different NGS data sets (ODT and TSEQ). Only the four most interesting gene selection methods are presented here. But, using a script in R that can be found in Additional file 2, one can also observe the results from the remaining methods.

**Figure 3:**
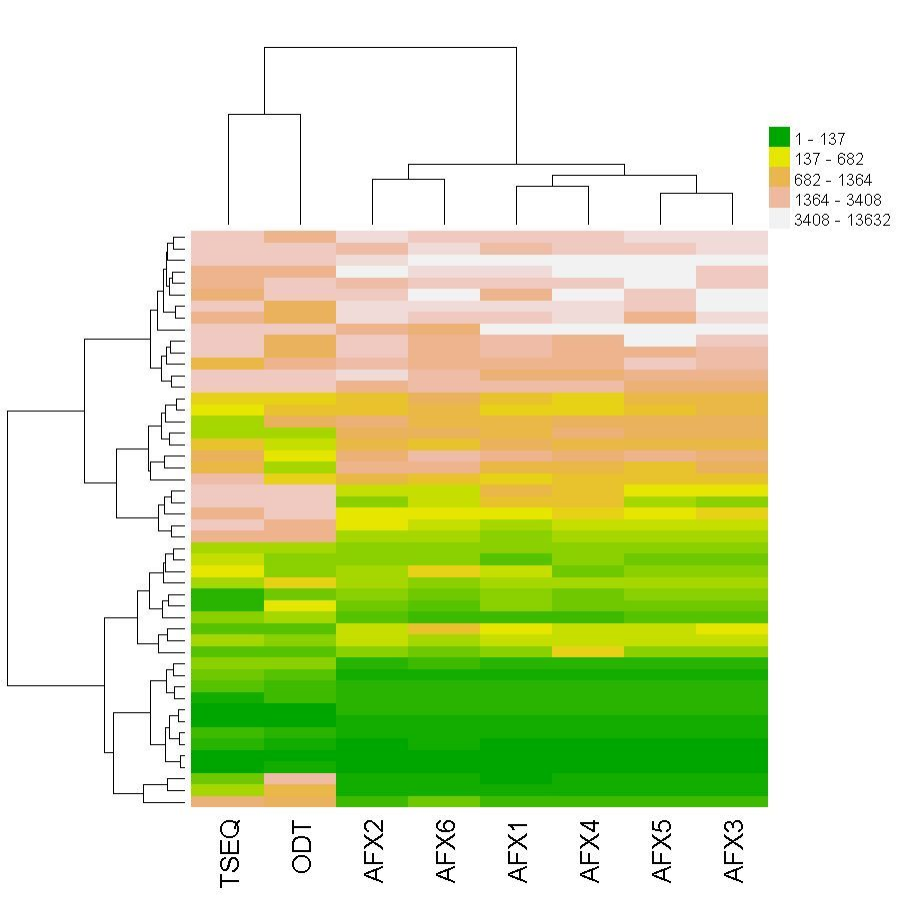
**Heat map of top ranked genes**. Heat map of the top 50 ranked genes using fold change gene selection where similarity of gene ranks is observed.

As already noted in the MAQC study, fold change gives the most stable results overall. One can notice that AFX1 provides a higher level of stability than ODT or TSEQ; however, POG for all data sets still lies above 80%. This is not the case for the second (Wilcoxon) and third (BaldiLong) most stable metrics where significantly lower POG scores were achieved, especially for top ranked genes. Again, microarrays outperformed NGS in terms of POG. There was actually only a single gene ranking method where results on microarrays were not the most stable, the Ebam (a mixture model gene-ranking technique proposed by Efron et al. [36]). However, Ebam belongs to a group of gene selection methods that all perform significantly worse than a simple fold change and are therefore of little use when high reproducibility is required. Due to better visualization, we only used one of six available affymetrix data sets in Figure 2. To demonstrate the high concordance of selected genes from different microarray test site data sets, a heatmap using fold change was produced in GeneSelector (Figure 3).

### Gene Set Enrichment Analyses

To compare the reproducibility of the two abovementioned platforms for gene expression measurements, classical GSEA was performed on six microarray data sets measured on Affymetrix platform and two different NGS data sets included in this study (Additional Files 3 and 4). The TSEQ and ODT enrichment results were compared with those of AFX1-AFX6 and also against each other for two different phenotypes, respectively.

Tables 3 and 4 present the top five gene sets with the highest NES from the TSEQ enrichment results and their corresponding ranks. They are compared with the AFX and ODT analysis results, considering NES. The AFX1-AFX6, as expected, showed very high levels of similarity between them and some differences when compared with TSEQ or ODT data. Gene sets from ODT gene set enrichment analysis had a negative NES, which indicates gene set enrichment at the bottom of a ranked list of genes and correlation with the opposite phenotype.

**Table 3:** Top 5 gene sets from TSEQ with corresponding ranks for enrichment in sample A

Gene set (phenotype A)	TSEQ	AFX1	AFX2	AFX3	AFX4	AFX5	AFX6	ODT (abs)
PENG_GLUTAMINE_DN	1	3	3	3	3	3	5	5
PENG_LEUCINE_DN	2	11	11	9	11	9	12	13
TARTE_PLASMA_BLASTIC	3	6	4	4	4	5	3	22
CHANG_SERUM_RESPONSE_UP	4	8	8	8	8	8	13	28
BHATTACHARYA_ESC_UP	5	5	7	7	6	7	7	7

**Table 4:** Top five gene sets from TSEQ with corresponding ranks for enrichment in sample B

Gene set (phenotype B)	TSEQ	AFX1	AFX2	AFX3	AFX4	AFX5	AFX6	ODT (abs)
CALCIUM_REGULATION_IN_CARDIAC_CELLS	1	2	2	2	2	2	2	1
HSA04020_CALCIUM_SIGNALING_PATHWAY	2	7	5	4	3	3	4	16
HSA04912_GNRH_SIGNALING_PATHWAY	3	4	4	3	6	4	7	20
HSA04740_OLFACTORY_TRANSDUCTION	4	5	3	6	5	7	5	2
HDACPATHWAY	5	10	7	7	8	8	10	3

A comparison of microarray-based gene expression analysis and NGS showed rather similar enrichment of the top gene sets, although the obtained results also suggest a non-negligible influence of the cDNA preparation method selection on result variability when using NGS.

### Meta-learning from GSEA Results

As already mentioned, this paper focuses on a novel method for analyzing GSEA results. To demonstrate the use of the proposed GSE-MLA methodology, we compared TSEQ and ODT against AFX1 and finally TSEQ and ODT against each other. Table 5 presents the stratified 10-fold cross-validation-based performance of the built decision trees. Even without the analysis of decision trees, one can see the difference between the compared platforms only by observing the ACC and AUC for the three comparisons. It is evident that both J48 and SimpleCART managed to build very accurate decision trees when microarrays were compared with NGS. On the other hand, they struggled when extracting knowledge from the TSEQ vs. ODT comparison as there are obviously very few gene sets that are significantly differently enriched in TSEQ and ODT in the bootstrapped GSEA runs.

**Table 5:** Results of GSE-MLA performance on three pairwise comparisons

	J48		SimpleCART		ADTree	
GSE-MLA Comparison	ACC	AUC	ACC	AUC	ACC	AUC
ODT vs. AFX1	89.50	92.31	91.00	88.98	100.00	100.00
TSEQ vs. AFX1	90.50	91.72	90.50	94.27	100.00	100.00
TSEQ vs. ODT	66.50	73.92	83.00	83.28	99.00	99.94

### Interpretation of Results

**Figure 4:**
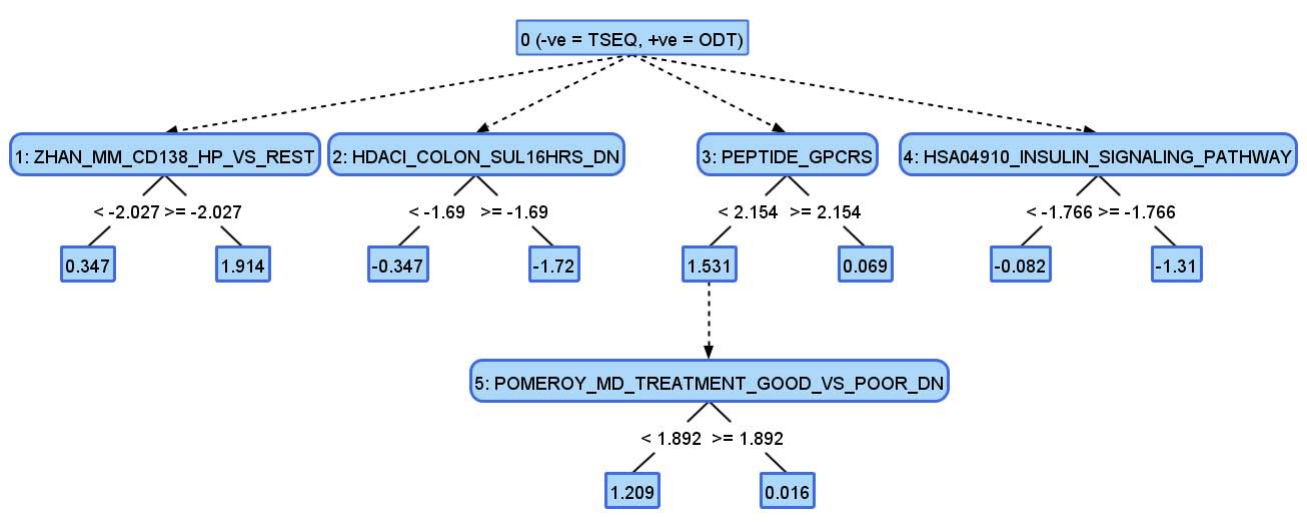
**Representation of GSE-MLA results using ADTree**. ADTree explaining the significant differences in gene set enrichment between ODT and TSEQ sample preparation.

From the biological point of view, the decision trees themselves are more interesting than their performance. The ADTree shown in Figure 4 demonstrates the gene sets that are differently expressed in ODT and TSEQ data preparation protocols. By this example, we illustrate another possible use of GSE-MLA analysis where two different preparation protocols for NGS are used and compared at the gene set enrichment level. There are two types of nodes in alternating decision trees-decision and prediction nodes. Our sample tree contains five decision nodes as a result of five boosting iterations used to build a tree. Twelve prediction nodes were used to assign weights to each sample to be classified. Positive samples indicate ODT preparation of samples and the negative ones represent TSEQ. All nodes in the first layer of the tree (numbered 1-4) have to be evaluated. Decision node number 5 was evaluated only for samples where expression of PEPTIDE_GPCRS was below 2.154. The sum of weights for all evaluated decision nodes represents the final answer-i.e., ODT for positive sums and TSEQ for negative weighted samples.

While one can already notice some of the differences in enrichment by comparing the overlaps of the most enriched gene sets, this example also demonstrates some co-enrichments that cannot be seen from a direct comparison-i.e., overlap of gene sets. As described above, only samples with PEPTIDE_GPCRS enrichment scores below 2.154 would also significantly differ in enrichment scores calculated for POMEROY_MD_PTREATMENT_GOOD_VS_POOR_DN.

Additionally, the gene sets shown in Figure 4 are of two different types, gene sets representing metabolic or signaling pathways and ones representing a chemical or genetic perturbation. The first group contains gene sets that are usually canonical representations of a specific biological process curated and compiled from several online pathway databases by domain experts [26], for example, HSA04910_INSULIN_SIGNALING_PATHWAY gene set containing 135 genes involved in the insulin-signaling pathway [37]; also to be mentioned is the PEPTIDE_GPCRS pathway with 75 genes, involved in the transduction of extracellular stimuli into intracellular signals [38].

The second group contains gene sets that represent gene expression signatures of genetic and chemical perturbations, each containing genes induced or repressed by a particular perturbation:

• HDACI_COLON_SUL16HRS_DN gene set with 72 genes which are down-regulated by sulindac, a trial nonsteroidal anti-inflammatory drug, potentially a chemopreventive agent for colon cancer, at specific conditions in SW260 colon carcinoma cells [39];

• ZHAN_MM_CD138_HP_VS_REST gene set containing 48 genes, the top ranked SAM (significance analysis of microarray)-defined overexpressed genes in CD138-enriched plasma cells for a subgroup of multiple myeloma patients [40]; and

• POMEROY_MD_PTREATMENT_GOOD_VS_POOR_DN gene set containing 24 genes highly associated with medulloblastoma treatment failure [41].

A collection of the remaining decision trees for results from Table 5 is available at the supplementary website.

## Discussion and Conclusions

GSE-MLA represents a novel approach to gene set enrichment analysis where reproducibility of results is observed at the pathway level. This paper demonstrates an effective way of uncovering the differences in enriched gene sets, comparing microarray and NGS experiments using decision tree-based knowledge extraction. Classic GSEA allows a comparison of different platforms by comparing NES or ranks of single gene set enrichments between platforms, whereas GSE-MLA also uncovers the hidden co-enrichments of gene sets from two compared data sets.

Additionally, this study demonstrates that one should be very careful when choosing a gene-ranking method for NGS data analysis. Even the simplest techniques like fold change give lower POG scores in comparison with microarray-based POG. Therefore, it is advised that one must consider specialized NGS gene-ranking methods tailored to count data instead of continuous gene expression values.

Classical gene set enrichment analysis shows only minor differences when TSEQ data are compared with AFX1-6 data. Relatively different enrichment levels in the top enriched gene sets between ODT and other data sets might also be contributed by a potential 3' bias in the ODT sample preparation procedure.

With the GSE-MLA results, one can notice that even the most enriched gene sets tend to suffer from instability of polarity-i.e., in some bootstrap samples, their value is extremely enriched in one direction and in some, in the opposite direction. Therefore it would also be possible to work with absolute values of NES and avoid the instability of NES polarity. There are still many ways to improve and extend the current GSE-MLA methodology. One of the promising possibilities is discretization of NES values to three (positive, negative, no enrichment) or two (positive, negative) classes.

## Availability and requirements

All data sets and source code (in R and Java) used for the experiments described in the paper are available at this supplementary website: http://ri.fzv.uni-mb.si/nextGene/sup. Java source code for GSE-MLA is also available in Additional File 5.

## Competing interests

The authors declare that they have no competing interests.

## Authors' contributions

GS and PK conceived and designed the method. GS and MB wrote the program and analyzed the data. GS and PK drafted the manuscript. All authors read and approved the final manuscript.

## Supplementary Material

Additional file 1 **Percentage of overlapping genes R code**. Source file of Figure 2. Click here for file

Additional file 2 **Heat map R code**. Source file of a heat map presented in Figure 3. Click here for file

Additional file 3 **GSE-MLA ODT results archive**. Comma separated values (CSV) files containing results of GSE-MLA for ODT data sets. Click here for file

Additional file 4 **GSE-MLA TSEQ results archive**. Comma separated values (CSV) files containing results of GSE-MLA for TSEQ data sets. Click here for file

Additional file 5 **GSE-MLA source code archive**. Java source code for GSE-MLA and visualization using decision trees. Click here for file
